# Differential gene expression in relation to mating system in Peromyscine rodents

**DOI:** 10.1002/ece3.5181

**Published:** 2019-04-16

**Authors:** Jesyka Meléndez‐Rosa, Ke Bi, Eileen A. Lacey

**Affiliations:** ^1^ Department of Integrative Biology University of California Berkeley California; ^2^ Museum of Vertebrate Zoology University of California Berkeley California; ^3^ Computational Genomics Resource Laboratory University of California Berkeley California

**Keywords:** gene expression, mating systems, monogamy, peromyscus, promiscuity, transcriptomics

## Abstract

Behaviors that increase an individual's exposure to pathogens are expected to have important effects on immunoactivity. Because sexual reproduction typically requires close contact among conspecifics, mating systems provide an ideal opportunity to study the immunogenetic correlates of behaviors with high versus low risks of pathogen exposure. Despite logical links between polygynandrous mating behavior, increased pathogen exposure, and greater immunoactivity, these relationships have seldom been examined in nonhuman vertebrates. To explore interactions among these variables in a different lineage of mammals, we used RNAseq to study the gene expression profiles of liver tissue—a highly immunoactive organ—from sympatric populations of the monogamous California mouse (*Peromyscus californicus*) and two polygynandrous congeners (*P. maniculatus* and *P. boylii*). Differential expression and co‐expression analyses revealed distinct patterns of gene activity among species, with much of this variation associated with differences in mating system. This tendency was particularly pronounced for MHC genes, with multiple MHC Class I genes being upregulated in the two polygynandrous species, as expected if exposure to sexually transmitted pathogens varies with mating system. Our results underscore the role of mating behavior in influencing patterns of gene expression and highlight the use of emerging transcriptomic tools in behavioral studies of free‐living animals.

## 1 INTRODUCTION

Determining how differences in gene expression affect and are affected by differences in behavior is essential to understanding the forces shaping organismal variation. The increasing availability of high‐throughput sequencing methods has facilitated the study of transcriptomic variation in nonmodel organisms by allowing the quantification of differences in gene expression in relation to complex behavioral phenotypes in natural populations of animals (Gleason & Burton, 2015; Todd, Black, & Gemmell, 2016). Mating systems, due to both their diversity and their critical role in shaping genetic variation in natural populations (Bessa‐Gomes, Legendre, & Clobert, 2004; Emlen & Oring, 1977; Selander, 1970; Wright, 1949), provide an ideal opportunity for exploring interactions between gene expression and evolutionarily important differences in behavior. Among mammals, mating systems range from monogamy to polygynandry, with numerous variants falling between these extremes (Clutton‐Brock, 2016,1989; Kleiman, 1977). Accordingly, comparative studies of gene expression in closely related species exhibiting different patterns of mating behavior promise to yield important insights into the transcriptomic causes and consequences of differences in mammalian behavior (Bengston et al., 2018; Munshi‐South & Richardson, 2017).

One component of the mammalian genotype that has been shown to be correlated with differences in mating systems is variability at major histocompatibility complex (MHC) genes, which code for proteins associated with the detection of and response to pathogens (Klein, 1986). Specifically, allelic diversity at MHC genes has been shown to be greater in polygynandrous compared to monogamous species (MacManes & Lacey, 2012; Sommer, Schwab, & Ganzhorn, 2002). This finding is thought to reflect increased exposure to sexually transmitted and other pathogens resulting from the greater number of reproductive partners per individual in polygynandrous species (Eames & Keeling, 2004). To date, efforts to examine correlations between mating systems and immunogenetic variation have focused primarily on nucleotide or allelic differences among individuals (MacManes & Lacey, 2012; Sommer et al., 2002). In contrast, relationships between mating systems and patterns of immunogene expression remain uncharacterized despite the expected importance of differences in gene activity to immune system function.

Mice of the genus *Peromyscus* provide an ideal system in which to explore relationships between mating system and expression of MHC and other immunogenes. Members of this genus are widely distributed in North America, occurring in habitats ranging from western coastal chaparral to eastern deciduous forest. Importantly, *Peromyscus* includes at least two evolutionarily independent origins of monogamy (Turner et al., 2010): the beach mouse (*P. polionotus*; Foltz, 1981) and the California mouse (*P. californicus;* Ribble, 1991). The latter species in particular has been shown to be both socially and genetically monogamous (Ribble, 1991; Ribble & Salvioni, 1990), meaning that individuals do not engage in extra‐pair matings that result in the production of offspring. Paternal care is essential to survival of neonates (Gubernick & Teferi, 2000; Gubernick, Wright, & Brown, 1993), underscoring the importance of at least socially monogamous relationships among adults. Along the coast of California, *P. californicus* occurs sympatrically with several closely related but polygynandrous members of this genus, providing unique opportunities for comparative studies of the genetic correlates of differences in mating systems.

To examine differences in immunogene expression as a function of variation in mating system, we compared patterns of expression in *P. californicus* to those in two polygynandrous species of *Peromyscus*. Specifically, we compared gene expression profiles in liver—an immunoactively important organ—for mice in co‐occurring populations of *P. californicus*, *P. maniculatus*, and *P. boylii*. We predicted that patterns of gene expression would differ between our polygynandrous and monogamous study species, with expression of immunologically active genes typically being greater in the polygynandrous species due to greater pathogen exposure and associated immune system activity. To provide a more robust test of this hypothesis, we replicated this comparison using mice from four distinct localities in California. To the best of our knowledge, this is the first study to explore relationships between expression of immunologically active genes and the mating systems of natural populations of mammals. In addition to generating important insights into the role of mating behavior in shaping this aspect of genetic variation, our analyses reveal new information regarding differences in gene expression in relation to complex behavioral phenotypes.

## 2 MATERIALS AND METHODS

### 2.1 Study species

We examined expression profiles for immunologically active genes in three species of *Peromyscus*. Two of these species, *P. maniculatus* and *P. boylii* (Birdsall & Nash, 1973; Kalcounis‐Rueppell & Spoon, 2009), are polygynandrous; the third, *P. californicus*, is both socially and genetically monogamous (Ribble, 1991). *Peromyscus maniculatus* is the most widespread and abundant Peromyscine in North America. This small‐bodied (10–24 g) species is an ecological generalist that occurs in a variety of habitats in California, including deciduous woodlands, deserts, coastal scrub, chaparral, and grasslands (Jameson & Peeters, 2004; King, 1968). *P. boylii* is somewhat larger (22–36 g) and is more ecologically restricted, occurring only in chaparral and scrub forest (Jameson & Peeters, 2004; King, 1968). *Peromyscus californicus,* the largest of the species examined (32–54 g), is also found in chaparral and scrub forest habitats from the San Francisco area to northern Baja California (Jameson & Peeters, 2004; King, 1968). Because these taxa co‐occur at multiple localities, immunogenetic expression can be compared for monogamous and polygynandrous species exposed to the same general suite of environmental conditions. Phylogenetically, *P. maniculatus* is more closely related to *P. californicus* than to *P. boylii* (Bradley et al., 2007). While this arrangement does not preclude possible impacts of phylogeny on our comparative analyses, inclusion of both *P. maniculatus* and *P. boylii* allows a more robust assessment of patterns of immunogenetic expression in polygynandrous species of mice.

### 2.2 Field sites

**Figure 1:**
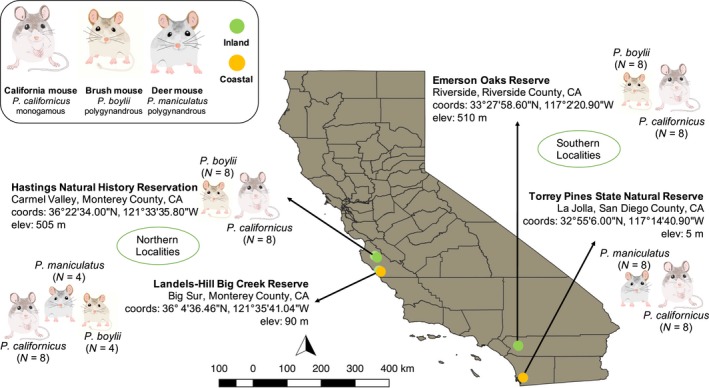
Field site localities in California and sampling regimes for the three species of Peromyscus examined. For each sampling locality, the species present are indicated, as is the number of adults sampled. Northern and southern sampling localities are indicated; coastal (orange) versus inland (green) localities are also identified

Mice were sampled at four localities, two in the northern and two in the southern portion of the range of *P. californicus*. From north to south, the distribution of this species is characterized by a pronounced rainfall gradient, with southern populations generally experiencing more arid conditions (California precipitation maps: www.cnrfc.noaa.gov); inclusion of populations from both extremes of the range of *P. californicus* encompassed habitat variation associated with this decline in rainfall. Similarly, because habitats differ markedly between the western and eastern sides of the coastal mountains in California (1981–2010 Climate data: www.cnrfc.noaa.gov), at each end of the north–south gradient sampled we selected one coastal and one inland population for analysis, resulting in a total of four sampling localities (Figure 1). At both coastal locations, *P. californicus* co‐occurs with *P. maniculatus*; at both inland locations, *P. californicus* co‐occurs with *P. boylii*. Thus, this sampling scheme allowed us to compare patterns of gene expression between monogamous and polygynandrous species across two axes of environmental variation.

### 2.3 Trapping and tissue sampling

All collection of tissue samples was completed between February and April 2016. At each sampling locality, animals were captured using Sherman live‐traps baited with rolled oats and containing a small ball of synthetic batting that the animals used as nesting material. A total of 180 traps per locality were set, with traps placed in pairs at 10 m intervals to create a grid measuring 150 m × 60 m and containing 90 trap stations (pairs of traps). At each sampling locality, traps were opened at 1600 hr and closed 3000 hr for 20 consecutive nights. Individuals captured were identified to species using standard pelage and body size characters (Jameson & Peeters, 2004). In addition, each animal was weighed, and its sex and reproductive status determined based on the appearance of the external genitalia.

To obtain liver tissue for use in transcriptomic analyses of gene expression (see below), at each sampling locality we collected four adult males and four adult females per species, resulting in liver tissue from a total of 64 individuals. Animals were euthanized via overdose with isoflurane followed by cervical dislocation. Immediately postmortem, samples (0.5 × 0.5 cm) of liver tissue were collected and placed in 1.5 ml of RNAlater. After soaking in RNAlater for 24 hr at 4°C, samples were frozen in liquid nitrogen until they could be transported to the Museum of Vertebrate Zoology on the UC Berkeley campus; once on campus, the samples were stored in a −80°C freezer until analysis. Additionally, we collected a sliver of skin (~ 5 × 2 mm) from the right pinna of each animal; mitochondrial sequences obtained from these samples were used to confirm species identifications based on external phenotypic traits.

All field work involving live mice was approved by the Animal Care and Use Committee at the University of California, Berkeley, and was consistent with the Guidelines for the Use of Wild Mammals in Research published by the American Society of Mammalogists (Sikes, 2016).

### 2.4 Genetic confirmation of species identity

Because the study species can be difficult to distinguish based solely on external pelage characteristics, we confirmed all species assignments made in the field using sequences from the mitochondrial cytochrome b (*cyt‐b*) locus. Genomic DNA was obtained from ear pinna samples from the 64 individuals sacrificed using a salt extraction protocol (Aljanabi & Martinez, 1997). PCR amplification of the entire 1140‐bp *cyt‐b* locus was performed for each individual using primers MVZ 05 and MVZ 16 (Smith & Patton, 1993). Our PCR master mix consisted of 25μL containing the following: 14.58 μl of ddH_2_O, 2.75 μl of 10× buffer (with MgCl_2_), 2.2 μl of MgCl_2_, 2 μl of Betaine, 1 μl of BSA (bovine serum albumin), 0.44 μl of dNTPs, 0.44 μl of each of the primers (forward and reverse), 0.15 μl of Taq polymerase (New England Bio Labs), and 1 μl of the DNA template. Amplification conditions consisted of an initial denaturation at 95°C for 4:00 min and 35 cycles of the following: denaturation at 95°C for 0:35 min, annealing at 49°C for 0:40 min, and extension at 72°C for 0:50 min. Our cycle sequencing mix consisted of 9 μl reactions containing the following: 5.94 μl of ddH_2_O, 1.43 μl of the forward primer (MVZ 05), 1.98 μl of the 5× Big Dye Buffer, 0.5 μl of Big Dye, and 1 μl of the PCR template. Sequences were edited and aligned using Geneious 7.1.7 (Kearse et al., 2012), after which each sequence was identified to species using the nucleotide BLAST feature in Genbank. For each sequence, species identity was based on the top BLAST sequence match (sequence identity > 98%) obtained for that sample.

### 2.5 Isolation of RNA

To quantify patterns of gene expression, we first isolated RNA from the liver samples collected during this study. A 15mg portion of each sample was homogenized using a PowerLyzer^®^ 24 with ceramic beads (MO BIO Laboratories, Inc.), after which RNA was extracted from the homogenized sample using the UltraClean^® ^Tissue and Cells RNA isolation kit (MO BIO Laboratories, Inc.). The concentration of each extract was determined using a NanoDrop (ThermoFisher); typically, extractions yielded ~ 200ng/μL of RNA. The quality of each extract was assessed using RNA 6000 Pico BioAnalyzer chips (Agilent); the mean RNA integrity number (RIN) score for our samples was 8 (range = 7.1–8.9). RNA extractions were then further purified using the DNase Max^®^ kit (MO BIO) or, for samples requiring additional concentration, RNeasy spin columns (Qiagen). Finally, samples were diluted to a standard concentration of 80 ng/μl in 25 μl of RNAase‐free water (total = 2000 ng RNA) for library preparation.

### 2.6 LIbrary preparation and transcriptomic sequencing

cDNA libraries were prepared using the Illumina^®^ platform KAPA Stranded mRNA‐Seq Kit (KAPA Biosystems), with the manufacturer's protocol modified to accommodate half reactions. For each sample, a total of 2μg of high‐quality RNA (RIN > 7) was suspended in 25 μl of RNase‐free water. Samples were fragmented at 94°C for 4 min in order to achieve a typical library insert size of 250 bp. We modified the manufacturer's protocol as follows: (a) First, to minimize artifacts resulting from over‐amplification and to maximize the number of unique fragments amplified, we divided the cleaned, adaptor‐ligated cDNA samples (step 10, KAPA technical data sheet) into two amplification reactions. Each of these sub‐reactions was amplified for 10 cycles, after which the reactions were pooled to produce a single final library per individual. (b) Second, the final, uniquely barcoded libraries were cleaned using low ratio SeraMag beads (Sigma‐Aldrich) instead of KAPA pure beads.

Initially, the size and concentration of each library were assessed visually by running samples on a 1% agarose gel that was then stained with ethidium bromide. More precise measures of library sizes were obtained using a BioAnalyzer DNA 1000 chip; mean library size (without primer sequences) was 264 (±13 bp). More precise measures of library concentrations were obtained using a Qubit (ThermoFisher) high‐sensitivity assay. Libraries measuring < 10 ng/μl were further concentrated using a 2× solid phase reversible immobilization (SPRI) bead cleanup procedure. A 10 μl aliquot of each final library was submitted to the Vincent J. Coates Genomics Sequencing Laboratory at the University of California, Berkeley, for sequencing. The 64 samples submitted were pooled equimolarly and then distributed across four lanes of Illumina HiSeq4000, 100PE sequencing.

### 2.7 Raw data processing

Raw sequence data were cleaned following the protocols of Singhal (2013) and Bi et al. (2012). In brief, adaptor contamination was removed from our fastq reads using cutadapt (Martin, 2011), after which Trimmomatic (Bolger, Lohse, & Usadel, 2014) was used to remove low‐quality reads (PHRED < 20). We then removed duplicate reads using Super‐Deduper (https://github.com/dstreett/Super-Deduper). Finally, bacterial contaminants were identified by aligning sequences in our data set to the *Escherichia coli *genome; bacterial sequences were then removed using Bowtie2 (Langmead & Salzberg, 2012).

### 2.8 Construction of species‐specific reference transcriptome assemblies

A reference genome for* P. maniculatus* was available from the NCBI database (GCF_000500345.1); the GFF assembly for this reference was downloaded and converted to GTF format using the gffread module in Cufflinks (Trapnell et al., 2010). In contrast, reference genomes were not available for *P. californicus* or *P. boylii*. Accordingly, to increase the efficiency and accuracy of mapping of reads in our data set and to reduce bias caused by mapping reads to a divergent genome, we used the LAST aligner (http://last.cbrc.jp/) to construct species‐specific reference transcriptome assemblies for these taxa. We selected one male and one female per species per study population and then concatenated the reads for all conspecifics. We indexed the *P. maniculatus* reference genome using the LAST aligner *lastdb* command, after which we used the LAST aligner *fastq‐interleave* module to merge the concatenated sequences for *P. californicus* and *P. boylii* (separate merge functions performed for each species) and to align the resulting clean reads for each species with the indexed *P. maniculatus* reference genome. Each aligned sequence was converted to sorted‐BAM format using SAMtools (Li et al., 2009). Alignments were sorted using *samtools sort* and indexed using *samtools index*. The *samtools mpileup* and *seqtk seq* functions (https://github.com/lh3/seqtk) were then used to convert alignments to FASTA format reference assemblies for each species. These reference transcriptomes were evaluated for completeness by comparing them to the *P. maniculatus* reference genome. If detected, missing data were replaced with the corresponding portion of the *P. maniculatus* genome. Ambiguous sites annotated with IUPAC ambiguity codes were replaced by randomly selecting one of the nucleotide bases (e.g., for Y, either C or T).

### 2.9 Read alignment, assembly, and quantification

Sequence reads for all 64 of our study animals were analyzed following the protocol of Pertea, Kim, Pertea, Leek, and Salzberg (2016). In brief, raw reads generated by our RNAseq analyses were mapped to the reference transcriptome for the associated species using HISAT2 (Kim, Langmead, & Salzberg, 2015). Mapped reads were then assembled into transcripts, and the expression level for each gene was estimated using StringTie (Pertea et al., 2016, 2015). While we identified novel transcripts as part of the Pertea et al. (2016) workflow, we only considered gene‐level abundance estimates during downstream analyses. Raw gene expression counts for each species were filtered to include only those loci annotated in all three study species. Raw counts were then imported to R and normalized using the “edgeR” trimmed mean of M‐values (TMM) method (Robinson, McCarthy, & Smyth, 2010). TMM‐normalized counts were filtered using counts per million (CPM); only genes with CPM > 1 in at least two individuals were retained for further analysis.

### 2.10 Weighted gene correlation network analyses

To examine relationships between patterns of gene expression (TMM‐normalized counts) and selected biological attributes (e.g., mating system), we first used a weighted gene correlation network analysis (WGCNA) approach (Langfelder & Horvath, 2008) to reveal subsets of loci that were characterized by similar expression profiles. Clusters of genes with similar expression profiles were identified by calculating Pearson correlation coefficients between gene expression counts and then raising these coefficients to a power of β. The value of β was determined by applying the approximate scale‐free topology criterion to all genes (Barabási & Bonabeau, 2003); network construction was conducted using β = 14 (scale‐free topology model fit, *R* ^2^ = 0.71). To determine whether the gene modules identified by this analysis were associated with selected biological attributes, we calculated Pearson correlation coefficients between module eigengenes (MEs; the first principle component axis of a given module) and each biological variable; the biological attributes considered were sex, habitat (coastal or inland), geographic region (north of south), and mating system (polygynandrous or monogamous). We then selected the module with the strongest positive correlation to mating system and plotted individual gene significance (GS) values versus module membership (MM). We extracted the top 30 most interconnected, or co‐expressed, genes within the module using their intramodular connectivity scores and visualized the connectivity patterns in VisANT (Hu et al., 2005). We examined potential functional associations among the genes within the module using the gene ontology (GO) categories and statistical overrepresentation tools in the PANTHER classification system (Thomas et al., 2003).

### 2.11 Differential expression and gene ontology enrichment

To examine expression profiles for individual loci, we performed a constrained correspondence analysis (CCA) on TMM‐normalized gene expression counts using the vegan package for R (Dixon, 2003). For each locus, differences in expression were assessed in relation to mating system, habitat, geographic region, and sex using the R package “edgeR” (Robinson et al., 2010). To examine the potential functional significance of genes that were differentially expressed in relation to these variables, we generated functional annotations (GO terms) for the *P. maniculatus* genome with “Blast2GO” (Gotz et al., 2008) using the full NCBI database. Prior to assessing whether loci that were differentially expressed tended to correspond to particular functional categories of genes (i.e., displayed GO category enrichment), we corrected for potential biases in gene length by calculating a probability weighting function (PWF) for the locus examined. We then used the R package “goseq” (Young, Wakefield, Smyth, & Oshlack, 2010) to assess potential GO category enrichment in our data set; the software package “GO.db” (Carlson, 2017) was used to summarize the results of these analyses. P values for these analyses were corrected for multiple comparisons using the Benjamini–Hochberg (BH) procedure.

In order compare patterns of expression at MHC genes across the study species, we filtered the TMM‐normalized counts for the MHC loci in our data set and then conducted a generalized linear model (GLM) DE analysis in “edgeR” with species as a treatment. To facilitate visual comparisons of expression profiles, we generated heatmaps of the TMM‐normalized count data using the R “gplots” heatmap.2 function (Warnes et al., 2016). Counts of gene expression were clustered on both the *y*‐axis (genes) and the *x*‐axis (individuals) based on similarity of expression profiles. Finally, we used the R package “pvclust” (Suzuki & Shimodaira, 2006) to assess the confidence of the hierarchical clusters using bootstrap resampling (10,000 iterations) and approximately unbiased (AU) p‐value estimations.

## 3 RESULTS

### 3.1 Molecular confirmation of species IDs

Analyses of *cyt‐b* sequences confirmed that all animals identified in the field as *P. californicus* (*n* = 32) and *P. boylii* (*n* = 16) were correctly assigned to these species. Of the 16 animals identified in the field as *P. maniculatus*, two males and two females from the Big Creek Reserve population (Figure 1) were identified genetically as *P. boylii*. As a result, the final sample size for each species was 32 for *P. californicus,* 20 for *P. boylii*, and 12 for *P. maniculatus;* the final data set for the Big Creek Reserve included transcriptomic data from all three study species.

### 3.2 RNA sequencing analyses

Each of our four Illumina HiSeq4000 lanes yielded ~390 M reads, resulting in a total of ~1,545 M reads in our data set. No samples failed our quality control thresholds, and thus, all reads were retained for subsequent analyses. Individuals aligned to their respective reference genomes with a mean of 84.4 ± 1.6% accuracy (range = 75%–87%). Across all four lanes of Illumina sequence, ~ 93% of nucleotides had Q scores > 30 (range = 87%–95%), indicating that the error rate for reading nucleotides was < 0.1%. The raw numbers of genes for which expression count data were available were as follows: 48,724 for *P. maniculatus*, 46,135 for *P. boylii*, and 58,605 for *P. californicus*. After filtering these data to include only genes detected in all three study species, a total of 20,625 loci were retained for analysis. Further filtering based on CPM resulted in a final count of 14,624 genes for analyses of gene expression profiles in relation to mating system.

### 3.3 WGCNA analyses

**Figure 2:**
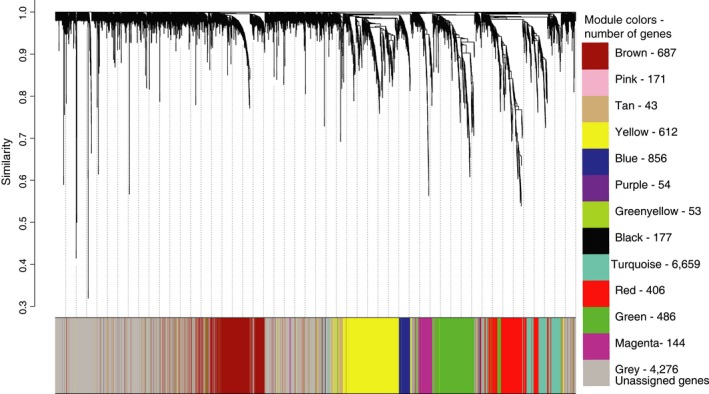
Hierarchical clustering of genes by expression profiles. Similarities in expression profiles for 14,624 genes were assessed via weighted correlation network analysis (WGCNA). Along the x‐axis, each terminal twig represents a distinct locus. For each locus, the y‐axis indicates the degree of similarity in expression across all samples examined; similarity was assessed using a topological overlap measure, with values approaching 0 indicating greater similarity in expression across samples. Loci were assigned to color‐coded modules based on similarities in gene expression, with genes that could not be assigned to any module placed in the gray cluster. A list of these color‐coded modules and the number of loci contained in each are provided at the right

**Figure 3:**
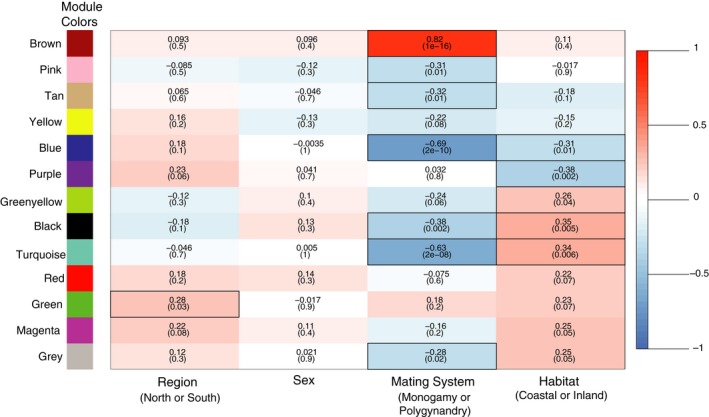
Relationships between gene expression and selected attributes of the animals sampled. Gene modules are indicated to the left of the table. Cells contain Pearson correlation coefficients between a module eigengene (first principal component for each gene module) and each of four potential predictors of gene expression; each predictor is identified at the bottom of the table; and the p‐value for each correlation is also shown. Red cells denote positive correlations, white cells denote no significant correlations, and blue cells denote negative correlations; color intensity denotes the relative strength of the correlation; and the scale for associated correlation coefficients is shown at right. Significant correlations (*p* < 0.05) are outlined with a black box

**Figure 4:**
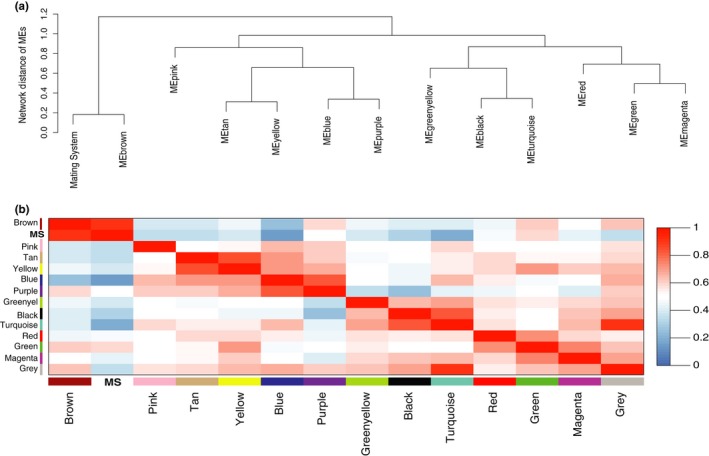
Relationships between gene expression and mating system. In (a), gene modules (MEs) are hierarchically clustered based on similarities in patterns of expression for the genes in each module; for the mating system variable, gene expression was assessed for the monogamous versus the polygynandrous study species. The y‐axis depicts the network distance between modules, with values closer to 0 indicating greater similarity between expression patterns in modules. In (b), a matrix of pairwise comparisons of module eigengene adjacency (connection strength), including the trait mating system (MS), is shown. Red cells denote high adjacency (positive Pearson correlations) between modules while blue cells denote low adjacency (negative correlations); white cells denote no significant correlation between modules. Color intensity denotes the relative strength of the correlation, as indicated in the color scale bar to the right

**Figure 5:**
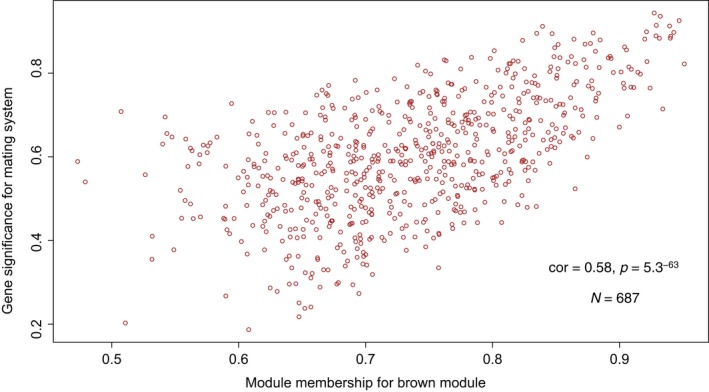
Relationships between gene significance for mating system and module membership for each of the 687 loci included in the brown gene module. Module membership (x‐axis) is a measure of how correlated the level of expression of each gene is with the module to which that gene was assigned; in this case the brown module. Gene significance (y‐axis) is a measure of how correlated each gene is with mating system; a gene significance of 0 would indicate that the gene is not significant with regard to mating system. Together, these measurements indicate that genes in the brown module are also correlated with mating system

Weighted gene correlation analyses (WGCNA) revealed 12 gene modules (loci sharing similar expression profiles) ranging in size from 43 to 6,659 loci per module; an additional 4,276 genes could not be assigned to a module, and these were placed in their own module (gray) and were not included in subsequent analyses (Figure 2). Pearson correlations between module eigengenes (MEs) and our target behavioral and environmental variables revealed a single module (brown) that was significantly positively associated with mating system (Figure 3). Hierarchical clustering of MEs also suggested that expression patterns within the brown module were strongly associated with mating system, as indicated by the magnitudes of correlation coefficients for this module (Figure 3) and the presence of a distinct cluster containing only the brown gene module and the mating system variable (Figure 4). Further, the significant positive correlation between gene significance (correlation between gene expression and mating system) and membership in the brown module (correlation between individual gene expression and the brown module) suggests a relationship between these loci and reproductive behavior (Figure 5). Visualization of interrelationships among the top 30 co‐expressed genes in the brown module revealed three highly connected genes with known immunological functions (Figure S1 in Appendix S1). Thus, multiple lines of evidence suggest that expression of genes in the brown module was associated with differences in mating systems.

Across all gene modules, the largest Pearson correlation coefficients detected were associated with mating system suggesting that, of the natural history parameters considered, patterns of gene expression were most strongly related to reproductive behavior. In contrast to the brown module, we identified six modules that were significantly negatively correlated with mating system (Figure 3). With regard to habitat (coastal vs. inland), three significant positive correlations and two significant negative correlations were identified; one significant positive correlation was identified for geographic region (north vs. south; Figure 3). No significant correlations were detected between gene modules and sex of the individuals sequenced (Figure 3). Two of the gene modules that were negatively associated with mating system (turquoise, black) were also significantly positively correlated with habitat; a third module (blue) was negatively associated with both mating system and habitat (Figure 3). In contrast, none of the gene modules that were significantly associated with mating system were also associated with geographic region (Figure 3). Thus, patterns of gene expression appeared to be more closely associated with mating system and habitat than the other environmental or biological parameters considered.

Examination of the individual genes assigned to the brown module (the only module that was positively correlated with mating system) revealed that of the 687 genes in this module, 164 (24%) could not be reliably identified with regard to function; these loci were excluded from further consideration. Functional classification of the remaining 523 genes in this module revealed that “binding” (31%) and “catalytic activity” (40%) were the most common molecular function GO categories associated with these loci, with “metabolic process” (23%) and “cellular process” (30%) as the most common biological process GO categories (Figure S2 in Appendix S1). Statistical overrepresentation tests indicated that the biological process GO categories “single‐organism metabolic process,” “cellular metabolic process,” and “metabolic process” were statistically overrepresented, while the categories “sensory perception,” “sensory perception of chemical stimulus,” and “sensory perception of smell” were underrepresented relative to a *Mus musculus* reference (Table 1). With regard to molecular function, the GO categories “catalytic activity” and “ion binding” were overrepresented relative to *Mus*, with no categories being underrepresented relative to this reference (Table 1).

**Table 1:** Results of overrepresentation tests of gene ontology (GO) category assignments for 523 genes in the brown expression module. For each GO category, both the expected and observed number of associated genes are indicated. Also indicated is whether observed values reflect under‐ or overrepresentation of that category, the associated enrichment values, and the Bonferroni correct p‐value for the comparison between observed and expected values (reference: *Mus musculus*). Results for GO biological processes are shown in (A); results for GO molecular processes are shown in (B)

GO term	No. of genes identified	Expected	Over/under	Fold enrichment	*p* value
GO: Biological Process					
Single‐organism metabolic process (GO:0044710)	101	63.49	+	1.59	1.36E−02
Cellular metabolic process (GO:0044237)	209	159.32	+	1.31	1.59E−02
Metabolic process (GO:0008152)	237	182.85	+	1.3	3.82E−03
Sensory perception (GO:0007600)	14	39.51	‐	0.35	1.13E−02
Sensory perception of chemical stimulus (GO:0007606)	2	31.22	‐	< 0.2	4.95E−08
Sensory perception of smell (GO:0007608)	1	24.79	‐	< 0.2	2.08E−06
Unclassified	9	37.72	‐	0.24	0.00E + 00
GO: Molecular function					
Catalytic activity (GO:0003824)	187	126.2	+	1.48	3.84E−06
Ion binding (GO:0043167)	162	118.17	+	1.37	1.57E−02
Unclassified	17	41.71	‐	0.41	0.00E + 00

### 3.4 Differential expression (DE) and gene ontology (GO) enrichment analyses

**Figure 6:**
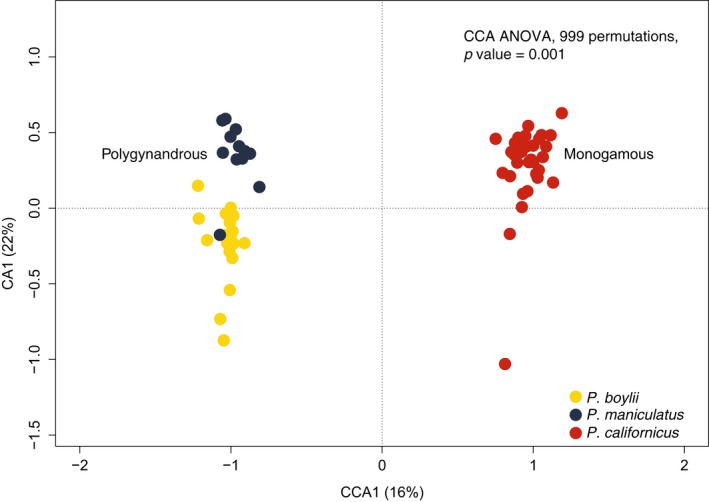
Differentiation of the study taxa based on patterns of gene expression. Data are from constrained correspondence analysis of normalized values of gene expression for each species with mating system included as the constraint. Each point represents an individual mouse (*N* = 64). The x‐axis shows the total amount of variation we can explain with mating system

Constrained correspondence (CCA) analyses revealed that patterns of gene expression clearly differed between the monogamous and the polygynandrous species examined (ANOVA permutation test; 999 permutations, *p* value = 0.001) (Figure 6). Of the 14,624 genes considered, 6,274 (42.9%) were differentially expressed between monogamous and polygynandrous species, 2,591 (17.7%) were differentially expressed between coastal and inland populations, 1,804 (12.33%) were differentially expressed between geographic regions, and 720 (4.9%) were differentially expressed between the sexes. Of the loci that were differentially expressed as a function of mating system, 3,106 (49.5%) were upregulated in the polygynandrous study species while the other 3,168 (50.5%) were upregulated in the monogamous *P. californicus* (Table 2). Pairwise analyses revealed that 7,256 genes were differentially expressed between *P. californicus* and *P. boylii*, 6,688 were differentially expressed between *P. californicus* and *P. maniculatus*, and 6,435 were differentially expressed between *P. maniculatus* and *P. boylii*. Differential expression analyses of the 30 most interconnected genes in the brown module revealed that all of these loci were upregulated in the monogamous species (Table S1 in Appendix S1). For all analyses of differential expression, the false discovery rate (FDR) was < 0.05.

**Table 2:** Twenty genes displaying the greatest difference in expression as a function of mating system. Negative logFC (log fold change) changes (shaded cells) indicate genes that were upregulated in the monogamous species; positive logFC changes indicate genes that were upregulated in the two polygynandrous species. Expression levels for each gene are provided as logCPM (log counts per million). *p* values were calculated using a Fisher's exact test and adjusted using the Benjamini–Hochberg “BH” procedure

Transcript ID	Gene ID	logFC	logCPM	*p* Value	FDR
XM_006978573.2	*Ascl3*	−12.51415017	4.163502689	4.10E−186	6.66E−183
XM_015999403.1	*trichosurin‐like*	−11.97155247	3.62193051	7.88E−173	6.07E−170
XM_006985611.2	*Stk32b*	−11.83483855	4.218310145	1.51E−185	2.21E−182
XM_016003647.1	*Gmnc*	−11.21877995	3.926310364	2.25E−178	2.06E−175
XM_006985706.2	*Acsm4*	−10.77462688	5.935853192	2.51E−200	1.22E−196
XM_006973109.2	*Rdh12*	−9.892254422	6.771920813	9.86E−189	2.06E−185
XM_016006175.1	*Ccdc57*	−9.657631018	10.53968832	3.61E−190	1.06E−186
XM_006981723.2	*Fbp2*	−9.55750474	8.031417925	1.16E−184	1.54E−181
XM_016006887.1	*Cndp1*	−9.49602698	7.551940644	9.80E−183	1.02E−179
XM_006983531.2	*Car15‐like*	−9.380107299	5.714159564	5.11E−175	4.40E−172
XM_006972591.2	*ST2A1‐like*	9.874701033	6.459581283	1.87E−184	2.28E−181
XM_006996847.2	*STD2‐like*	10.21728467	4.942431264	2.26E−174	1.84E−171
XM_015990843.1	*CYP3A5‐like*	10.26160713	5.329011684	8.87E−179	8.65E−176
XM_015989344.1	*CYP3A6‐like*	10.3095931	5.880682064	6.37E−188	1.16E−184
XM_015991544.1	*PLA2s‐like*	10.55658689	5.765298083	4.59E−189	1.12E−185
XM_006982472.2	*Mybph*	10.69646589	5.024975905	4.42E−183	4.97E−180
XM_016008782.1	*Serum albumin‐like*	11.08331147	5.62396081	2.88E−194	1.05E−190
XM_006997575.2	*SULT2A1‐like*	11.41561584	4.233025924	2.88E−172	2.11E−169
XM_006972604.2	*SULT2A1‐like*	13.60854338	6.190396867	5.35E−224	3.91E−220
XM_006977680.2	*Spink1*	15.03855013	6.683616419	1.97E−240	2.89E−236

Gene ontology enrichment analyses revealed that the GO cellular categories “cellular component” and “MHC class I protein complex” were enriched in the polygynandrous study species (Table 3). Similarly, the GO process category “biological process” and the molecular function categories “organelle membrane,” “heme binding,” “molecular function,” and “calcium ion binding” were enriched in these species (Table 3). In contrast, the cellular GO categories “cell surface” and “extracellular region” and the biological process categories “proteolysis” and “G‐protein coupled receptor signaling pathway” were enriched for the monogamous *P. californicus* (Table 3). Thus, there were clear differences in the functional subsets of genes that were differentially expressed in relation to mating system. In contrast, the “MHC class I protein complex” category of genes was not differentially expressed between the two polygynandrous species examined (Table S3 in Appendix S1).

**Table 3:** Enrichment of gene ontology (GO) categories of genes differentially expressed in relation to mating system. Data are from 6,274 genes that were differentially expressed between the monogamous and the two polygynandrous study species. GO categories examined were cellular component, biological process, and molecular function. In all cases, enrichment was significant at *p* < 0.05 after correction with the Benjamini–Hochberg “BH” procedure. Shaded entries denoted GO categories containing genes that were enriched in both mating systems

Upregulated in polygynandrous		Upregulated in monogamous	
Cellular component		Cellular component	
GO ID	Term	GO ID	Term
GO:0,005,578	Proteinaceous extracellular matrix	GO:0,005,578	Proteinaceous extracellular matrix
GO:0,005,887	Integral component of plasma‐ membrane	GO:0,005,887	Integral component of plasma‐ membrane
GO:0,005,615	Extracellular space	GO:0,005,615	Extracellular space
GO:0,016,021	Integral component of membrane	GO:0,016,021	integral component of membrane
GO:0,005,575	Cellular component	GO:0,009,986	Cell surface
GO:0,042,612	MHC class I protein complex	GO:0,005,576	Extracellular region
**Biological process**		**Biological process**	
GO:0,008,150	Biological process	GO:0,006,508	Proteolysis
**Molecular function**		GO:0,007,186	G‐protein coupled receptor signaling pathway
GO:0,031,090	Organelle membrane		
GO:0,020,037	Heme binding		
GO:0,003,674	Molecular function		
GO:0,005,509	Calcium ion binding		

### 3.5 Differential expression of MHC genes

**Figure 7:**
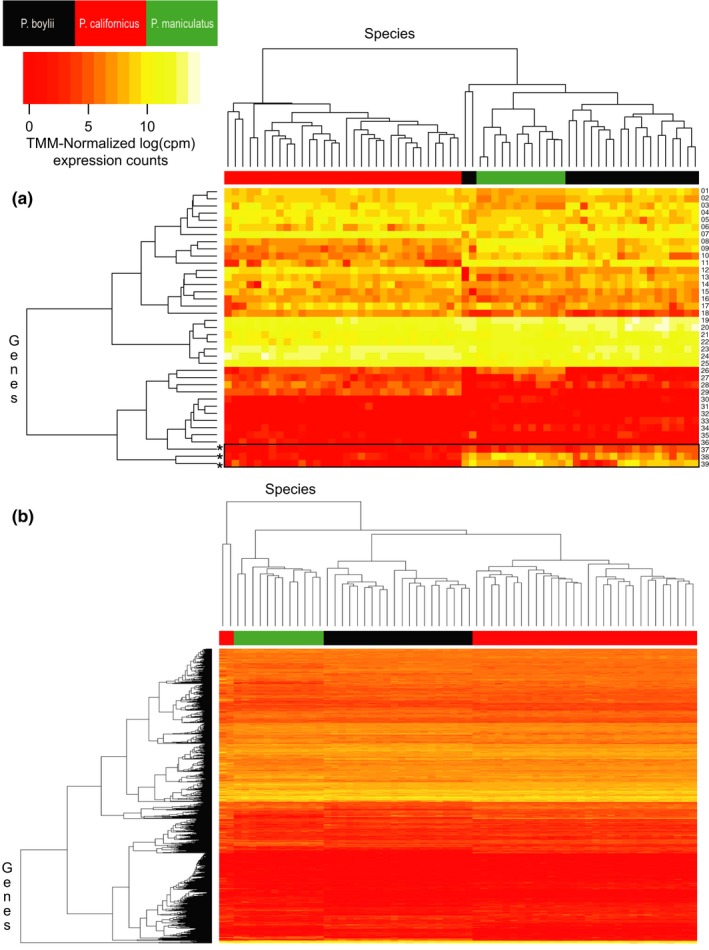
Comparisons of gene expression levels for (a) MHC loci (*N* = 39) and (b) all genes examined (*N* = 14,624). Measures of expression are TMM‐normalized logCPM (log counts per million); the scale for expression counts is shown in the upper left corner. In both panels, the x‐axis is a dendrogram that clusters expression data by similarity between individuals; color coding for species is shown in the upper left. The y‐axis clusters genes by similarities in expression profiles. The three loci showing increased expression in the polygynandrous species—relative to monogamous species—are indicated with asterisks at the bottom left of panel A. Gene descriptions for the numbered genes in panel A can be found in Table S2 of the supporting information Appendix S1

**Figure 8:**
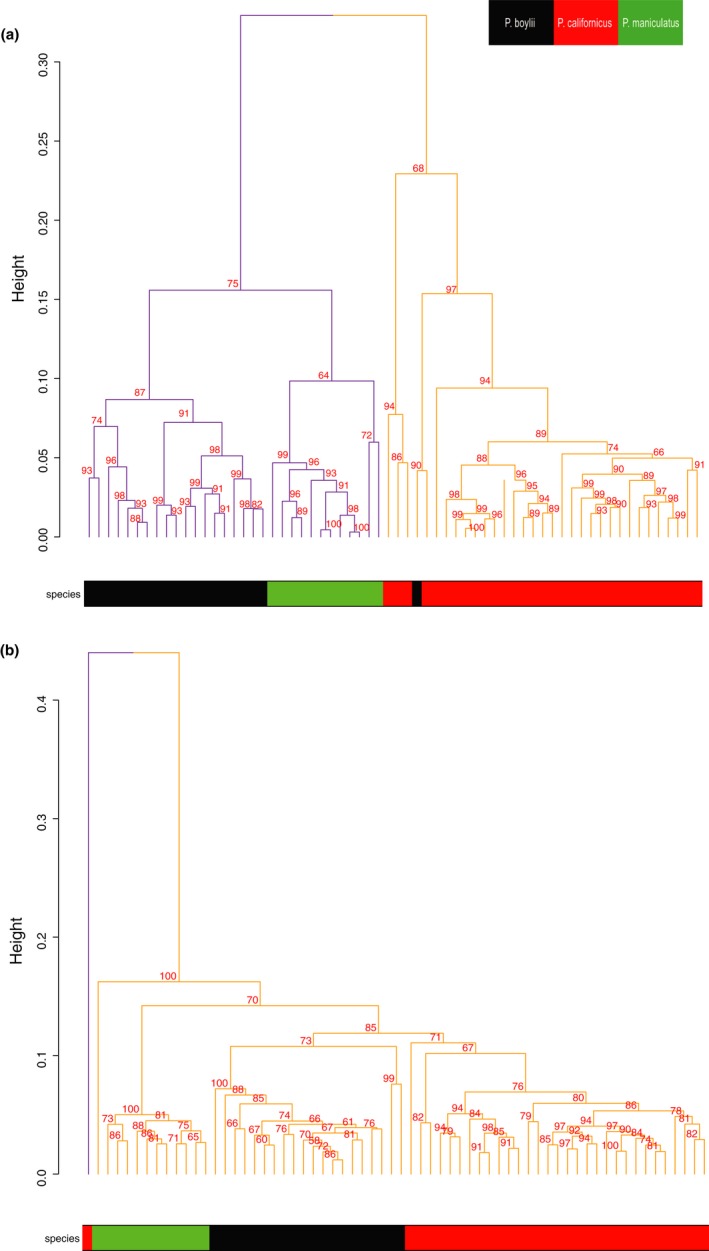
Hierarchical cluster dendrogram of individuals based on (a) MHC gene expression and (b) liver transcriptome expression. Approximately unbiased (AU) *p*‐values (%) are provided for each node. AU *p*‐values are calculated via multiscale bootstrap resampling (10,000 iterations), and an AU *p*‐value over 95% indicates cluster support at a 0.05 significance level. Distinct clusters are shown with different colors. The y‐axis measures the distance between the clusters as “height.” Species are indicated using a colored bar on the x‐axis, and a legend is provided in the top right corner

**Figure 9:**
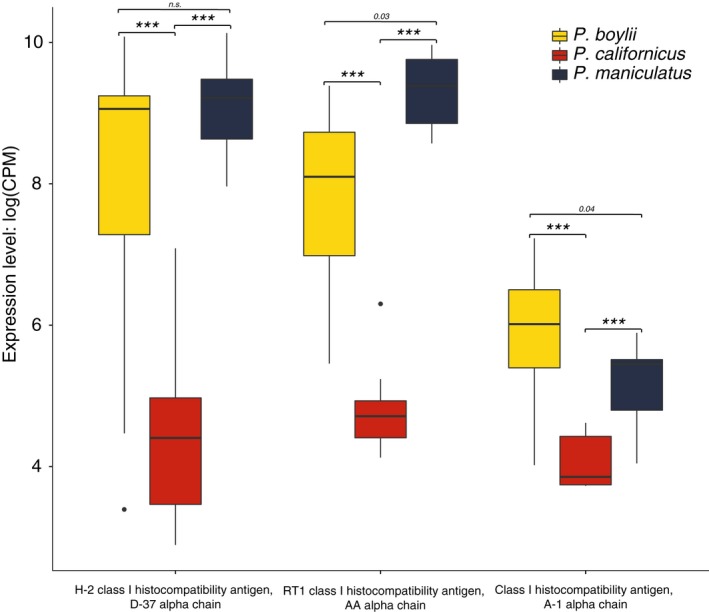
Box plot of expression levels as logCPM (log counts per million)—transcript counts per million—for the three most differentially expressed MHC genes. Kruskal–Wallis tests for each gene were significant *p* < 0.001, with *Peromyscus maniculatus* and *Peromyscus boylii* displaying significantly higher expression levels relative to *Peromyscus californicus* for all three genes (Dunn's Test; all *p* < 0.001, holm corrected)

After filtering TMM‐normalized count expression data to identify MHC Class I and Class II genes, a total of 46 MHC loci were retained for analysis. Of these, 39 were differentially expressed among the three study species (FDR < 0.05). Hierarchical clustering of individuals revealed a pronounced difference in MHC expression profiles between mating systems, with the monogamous *P. californicus* appearing distinct from either of the polygynandrous study species (Figure 7a). Assessment of this clustering via bootstrapping and approximately unbiased (AU) p‐value estimation generated two main clusters of animals that corresponded closely to mating system type; although visually distinct, these clusters were not statistically different (AU *p*‐value < 0.95) (Figure 8a). In general, while the “MHC class I protein complex” was upregulated in the polygynandrous species (Table 3), expression levels for individual MHC genes did not follow the predicted pattern of greater expression in the polygynandrous species examined. Three MHC genes, however, displayed particularly pronounced differences as a function of mating system (where polygynandrous > monogamous): one H2 Class I histocompatibility D37 α chain coding gene and two Class I histocompatibility A antigen α chains (Figures 7and 9). Interestingly, we did not find differences in expression at the DQα locus—a common target in previous MHC studies (see gene 04 in Figure 7a; gene ID XM_006997389.2 in Table S2 of supporting information Appendix S1).

The tendency for MHC gene expression profiles to cluster by mating system did not, however, extend to more inclusive, transcriptome‐level measures of gene expression (Figure 7b). Clustering analyses of our transcriptome data (immune and nonimmune genes) revealed strong support (AU *p*‐value > 0.95) for a single large cluster of individuals that included members of all three study species (Figure 8b), with only a single *P. californicus* falling outside of this cluster. Thus, the association between gene expression and mating system appeared to be more salient for MHC loci, although increased sample sizes would improve our ability to evaluate these relationships statistically.

## 4 DISCUSSION

Our analyses indicate that gene expression profiles in liver tissue differed significantly as a function of mating system, with distinct functional categories of genes being upregulated in the monogamous *P. californicus* compared to the polygynandrous *P. maniculatus* and *P. boylii*. This association with mating system was more pronounced than associations between gene expression and either habitat type (inland vs. coastal) or geographic region (northern vs. southern populations), suggesting that mating system was the best predictor of differences in liver gene expression in our study species. In particular, MHC genes tended to be upregulated in the two polygynandrous study species, as expected if members of these species are exposed to a greater number of pathogens due to their greater contact with conspecifics. These analyses differ from previous efforts to explore relationships between mating systems and MHC variation in that they (a) consider expression profiles rather than nucleotide or allelic variation, (b) include numerous expressed MHC loci, and (c) encompass replicate pairs of co‐occurring monogamous and polygynandrous populations of *Peromyscu*s. Accordingly, these analyses provide the strongest evidence to date that mating systems impact patterns of immunogenetic variation in natural populations of mammals.

Although our analyses revealed multiple lines of evidence indicating that mating system is an important determinant of gene expression profiles, the potential effects of other factors cannot be excluded. In particular, our data set does not allow us to distinguish fully between the effects of mating system and species identity. Future studies will benefit from including analyses of the monogamous *P. polionotus* and one or more of its sympatric, polygynandrous congeners. Given that *P. polionotus* and *P. californicus* appear to represent independent origins of monogamy within this genus (Turner et al., 2010), inclusion of these additional species will generate an important replicate data set regarding relationships between mating system and gene expression. Thus, while additional studies are needed to confirm the contribution of mating system to patterns of gene expression, our findings provide compelling evidence that expression of multiple genes, including functionally important immunogenes, differs between monogamous and polygynandrous species.

### 4.1 Comparisons with previous studies

Previous analyses of MHC variation in *Peromyscus* have typically examined allelic‐ or nucleotide‐level variation at one to a few MHC genes. These studies report high levels of DNA and protein‐level diversity for MHC Class II loci in *P. maniculatus* (Richman, Herrera, & Nash, 2001, 2003). Particularly relevant is work by MacManes and Lacey (2012), who compared variation at the MHC Class II DQα locus in *P. californicus* and *P. maniculatus* collected at the Big Creek Reserve, one of the sampling sites examined here. Their analyses revealed greater allelic‐ and nucleotide‐level variation as well as stronger evidence for selection on the DQα locus in the polygynandrous *P. maniculatus*. Bacterial diversity in vaginal swabs from females was also greater for *P. maniculatus* than for *P. californicus* (MacManes, 2011), leading MacManes and Lacey (2012) to conclude that differences between the mating systems of these species were associated with differences in exposure to sexually transmitted pathogens that likely contributed to enhanced variability at the DQα locus. Although we did not find differences in expression at the DQα locus, our results—which are based on samples from multiple populations and analyses of a much larger number of loci—are generally consistent with this conclusion. Thus, our analyses serve to expand the generality of previous findings regarding relationships between mating system and immunogenetic variability in the genus *Peromyscus*.

Our analyses also differ from previous studies by focusing on patterns of gene expression; to the best of our knowledge, ours is one of the first studies to assess differences in MHC expression as a function of mating system in natural populations of animals. Expression profiles at MHC genes clearly differed between our monogamous and polygynandrous study species. In contrast, the distinctions between expression profiles for the two polygynandrous species were less pronounced. This outcome is intriguing given that *P. californicus* and *P. maniculatus* are more closely related to each other than to *P. boylii* (Bradley et al., 2007) and given that *P. maniculatus* and *P. boylii* tend to occupy coastal versus inland habitats, respectively. Accordingly, the absence of pronounced differences in MHC gene expression between the latter two species underscores the apparent role of mating system in shaping the expression profiles of the study taxa. Future studies that explore this relationship in greater detail should generate important insights into interactions among behavioral phenotypes and gene function (Bengston et al., 2018).

### 4.2 Functional implications of differences in immunogene expression

Genes identified here as being highly correlated with mating system included a variety of immune‐related loci. For example, MHC Class I genes that code for proteins responsible for the binding and presentation of foreign antigens to the immune system (Paulsson, 2004) were upregulated in the two polygynandrous species examined. Further, the cellular component GO category “MHC class I protein complex” and the molecular function GO categories “calcium ion binding” and “heme binding” were enriched for genes upregulated in the polygynandrous species. Both calcium and heme play a variety of interesting and essential roles in immune system processes; Ca^++^ is involved in controlling antibody formation (Diamantstein & Odenwald, 1974), and heme in the activation of innate immune receptors (Dutra & Bozza, 2014) and the starving of microbes (Nairz et al., 2018). In contrast, all of the most interconnected genes in the brown module were upregulated in the monogamous species, including genes associated with removal of foreign biotic materials from cells (*mdr1* locus; Ho, Moodie, & Satsangi, 2003; Hoffmann & Kroemer, 2004) and metabolizing toxic compounds (*P450 2C11* or *CYP2C11* locus; Nebert, & Gonzalez, 1987). This difference in the functional gene categories that were upregulated in the polygynandrous versus the monogamous study species raises the intriguing possibility that how animals respond to immune challenges differs with mating system. Studies that explore the specific functional roles of genes that are up‐ versus downregulated in relation to mating system should generate new insights into the selective pressures acting on immunologically active genes.

### 4.3 Factors affecting immunogenetic diversity

The data presented here suggest that mating systems can impact patterns of gene expression in free‐living populations of mammals. Although our analyses identified mating system as the most important predictor of differences in gene expression among our study animals, we cannot exclude the possibility that other behavioral, ecological, and demographic traits are also important. For example, population density may impact pathogen exposure, with subsequent effects on immune system genetics. In European bison, higher population density is associated with increased nematode infection (Radwan et al., 2010) and it is likely that this increase in active infections leads to increased expression, selection, and diversity of MHC genes over time. As such, studies of behaviorally similar species of *Peromyscus* that differ with regard to key demographic, ecological, or life history traits (e.g., population density, mesic vs. desert habitats, litter size) can be used to explore the effects of the latter parameters on gene expression. By combining comparative field data from phylogenetically appropriate subsets of congeners with analyses of transcriptomic variability, it should be possible to generate a considerably more comprehensive understanding of how different aspects of the biology of natural populations of vertebrates contribute to differences in immunogenetic variation at the nucleotide, allelic, and gene expression levels.

## AUTHOR CONTRIBUTIONS

Meléndez‐Rosa designed and performed the research; Bi contributed analytical tools; Meléndez‐Rosa and Bi analyzed the data; and Meléndez‐Rosa and Lacey wrote the paper.

### DATA AVAILABILITY STATEMENT

BAM files and associated references are available on the NIH SRA data repository (BioProject: PRJNA508392). *P. maniculatus* functional GO annotations and additional metadata are available on the Dryad Digital Repository: https://doi.org/10.5061/dryad.9883b80.
