# Supplementary material for: Differential gene expression in relation to mating system in Peromyscine rodents
Source: Ecol Evol. 2019 Apr 16;9(10):5975–90. doi: 10.1002/ece3.5181 (PMC6540711; doi:10.1002/ece3.5181)
Supplement: Supplementary file 1 [file ECE3-9-5975-s001.docx]

Supplementary Materials: Differential gene expression in relation to mating system in Peromyscine rodents

Jesyka Meléndez-Rosa^1,2,*^, Ke Bi^2,3^, and Eileen A. Lacey^1,2^

^1^Department of Integrative Biology, University of California, Berkeley

^2^Museum of Vertebrate Zoology, University of California, Berkeley

^3^Computational Genomics Resource Laboratory, University of California, Berkeley

^*^Correspondence: Museum of Vertebrate Zoology, Valley Life Sciences Bldg., 3101, UC Berkeley Rd, Berkeley, CA 94720; (510) 642-3567; jesykamelendez@gmail.com

| **Table S1.** Differential expression values for the top 30 most interconnected genes in the brown module. Negative logFC changes are associated with genes up-regulated in the monogamous species. Positive logFC changes are associated with genes up-regulated in promiscuous species. P values were calculated using a Fisher’s exact test and adjusted using the Benjamin-Hochberg ‘BH’ procedure. | | | | | |
| --- | --- | --- | --- | --- | --- |
| **Transcript ID** | **Gene ID** | **logFC** | **logCPM** | **PValue** | **FDR** |
| XM_006978573.2 | *Ascl3* | -12.51415017 | 4.163502689 | 4.10E-186 | 6.66E-183 |
| XM_006974689.1 | *Cpa6* | -10.53408024 | 3.088856123 | 9.62E-160 | 5.41E-157 |
| XM_006981723.2 | *Fbp2* | -9.55750474 | 8.031417925 | 1.16E-184 | 1.54E-181 |
| XM_006983531.2 | *Car15* | -9.380107299 | 5.714159564 | 5.11E-175 | 4.40E-172 |
| XM_006972172.2 | *Slc23a3* | -8.894238904 | 4.896328762 | 1.38E-159 | 7.47E-157 |
| XM_006992342.2 | *Tnfrsf4* | -5.746089118 | 7.447908024 | 2.74E-95 | 2.97E-93 |
| XM_006975345.2 | *C6orf163* | -5.39246226 | 2.42900471 | 4.41E-81 | 3.43E-79 |
| XM_006997671.2 | *P450 2C11* | -4.382513853 | 5.961800086 | 1.28E-63 | 6.07E-62 |
| XM_015993516.1 | *Bhlhe22* | -4.336443037 | 3.722025608 | 2.01E-61 | 8.87E-60 |
| XM_016003412.1 | C2orf16 | -4.266745559 | 3.45557102 | 6.59E-60 | 2.77E-58 |
| XM_006970720.2 | *B3gnt4* | -4.124444461 | 0.847005815 | 1.58E-49 | 4.94E-48 |
| XM_006971211.2 | *NXPE Family* | -3.985611367 | 5.281748929 | 6.24E-55 | 2.32E-53 |
| XM_015986321.1 | *ZNF558* | -3.535982899 | 3.478397521 | 3.64E-45 | 9.66E-44 |
| XM_015995536.1 | *P450 2C11* | -3.504134826 | 5.150001495 | 1.21E-44 | 3.14E-43 |
| XM_016008732.1 | *mdr1* | -3.461220304 | 7.372420897 | 7.07E-44 | 1.79E-42 |
| XM_006995193.2 | *Ints5* | -3.350488693 | 7.405691302 | 1.27E-41 | 2.99E-40 |
| XM_015986857.1 | *OR52I2* | -3.346883715 | 1.297955367 | 9.42E-39 | 2.00E-37 |
| XM_016009124.1 | *ZNF558* | -2.908923423 | 2.474884354 | 5.14E-32 | 8.72E-31 |
| XM_006983535.2 | *Dgcr14* | -2.494902278 | 5.793058596 | 1.83E-25 | 2.45E-24 |
| XM_006995102.2 | *NipSnap* | -2.458101375 | 5.284847773 | 8.57E-25 | 1.13E-23 |
| XM_006984434.2 | *Fbxo30* | -2.331948831 | 5.598506749 | 1.06E-22 | 1.29E-21 |
| XM_006970744.2 | *Ift81* | -2.262612649 | 3.657402353 | 1.91E-21 | 2.19E-20 |
| XM_006990762.2 | *Kiaa1462* | -2.196671879 | 5.683378837 | 1.63E-20 | 1.79E-19 |
| XM_006979323.2 | *Trip4* | -2.168159392 | 4.965032856 | 4.83E-20 | 5.21E-19 |
| XM_006979967.2 | *ZNF58* | -2.022307159 | 3.448117289 | 1.04E-17 | 1.03E-16 |
| XM_006985016.2 | *Dpy19l3* | -1.892339449 | 6.151204299 | 6.17E-16 | 5.59E-15 |
| XM_015986989.1 | *Ids* | -1.879164337 | 5.081030136 | 1.00E-15 | 8.97E-15 |
| XM_006970320.2 | *ADH6* | -1.554787946 | 8.381083228 | 1.87E-11 | 1.33E-10 |
| XM_006995928.2 | *Gtpbp3* | -1.492436255 | 4.086956682 | 1.23E-10 | 8.34E-10 |
| XM_006991134.2 | *Sdhaf4* | -1.40552123 | 4.578310835 | 1.19E-09 | 7.56E-09 |

| **Table S2.** Gene descriptions for the 46 MHC Class I and II genes (including MHC regulatory and transactivator protein coding genes) analyzed for differential expression. The column ‘Figure 7A number ID’ refers to the number IDs in Figure 7A; entries containing an ‘n/a’ in this column were not differentially expressed. | | |
| --- | --- | --- |
| **Figure 7A**  **number ID** | **Transcript ID** | **Gene Description** |
| 01 | XM_006997044.2 | Class II histocompatibility antigen, M beta 1 chain |
| 02 | XM_006997045.2 | Class II histocompatibility antigen, M alpha chain |
| 03 | XM_006998788.1 | H-2 class I histocompatibility antigen, Q9 alpha chain-like |
| 04 | XM_006997389.2 | H-2 class II histocompatibility antigen, A-R alpha chain-like (DQα) |
| 05 | XM_006998677.2 | H-2 class I histocompatibility antigen, Q10 alpha chain-like |
| 07 | XM_006997392.2 | H-2 class II histocompatibility antigen, E-K alpha chain |
| 06 | XM_015991363.1 | H-2 class I histocompatibility antigen, Q10 alpha chain-like |
| 08 | XM_006997281.2 | Class I histocompatibility antigen, Non-RT1.A alpha-1 chain-like |
| 09 | XM_006998564.2 | H-2 class I histocompatibility antigen, Q10 alpha chain-like |
| 10 | XM_006998845.2 | H-2 class I histocompatibility antigen, Q9 alpha chain-like |
| 11 | XM_006998064.2 | H-2 class I histocompatibility antigen, Q10 alpha chain-like |
| 12 | XM_006997391.2 | H-2 class II histocompatibility antigen, E-D beta chain-like |
| 13 | XM_006997393.2 | HLA class II histocompatibility antigen, DRB1-4 beta chain-like |
| 14 | XM_006997038.2 | rano class II histocompatibility antigen, A beta chain-like (DQβ) |
| 15 | XM_006997388.2 | rano class II histocompatibility antigen, A beta chain-like (DQβ) |
| 16 | XM_016002443.1 | Class II, major histocompatibility complex, transactivator (*Ciita*) |
| 17 | XM_015991686.1 | H-2 class I histocompatibility antigen, Q9 alpha chain-like |
| 18 | XM_016004093.1 | Major histocompatibility complex class I-related gene protein |
| 19 | XM_006996817.2 | H-2 class I histocompatibility antigen, Q10 alpha chain-like |
| 20 | XM_015990931.1 | H-2 class I histocompatibility antigen, Q10 alpha chain-like |
| 21 | XM_006998077.2 | H-2 class I histocompatibility antigen, Q10 alpha chain-like |
| 22 | XM_015990461.1 | H-2 class I histocompatibility antigen, Q10 alpha chain-like |
| 23 | XM_015988730.1 | H-2 class I histocompatibility antigen, Q10 alpha chain-like |
| 24 | XM_006985129.2 | CD74 molecule (Cd74) |
| 25 | XM_015990720.1 | H-2 class I histocompatibility antigen, L-D alpha chain-like |
| 26 | XM_015991212.1 | patr class I histocompatibility antigen, A-126 alpha chain-like |
| 27 | XM_006997049.2 | HLA class II histocompatibility antigen, DP beta 1 chain-like |
| 28 | XM_006997048.2 | HLA class II histocompatibility antigen, DO alpha chain |
| 29 | XM_015991681.1 | H-2 class I histocompatibility antigen, Q9 alpha chain-like |
| 30 | XM_006996075.2 | HLA class I histocompatibility antigen, A-11 alpha chain-like |
| 31 | XM_006997706.2 | HLA class I histocompatibility antigen, A-25 alpha chain-like |
| 32 | XM_015989452.1 | HLA class I histocompatibility antigen, A-25 alpha chain-like |
| 33 | XM_015989455.1 | Class I histocompatibility antigen, B alpha chain-like |
| 34 | XM_015990242.1 | HLA class II histocompatibility antigen, DP alpha 1 chain |
| 35 | XM_006996070.1 | Saoe class I histocompatibility antigen, A alpha chain-like |
| 36 | XM_006996073.2 | HLA class I histocompatibility antigen, A-24 alpha chain-like |
| 37 | XM_015989456.1 | Class I histocompatibility antigen, A-1 alpha chain-like |
| 38 | XM_015989910.1 | RT1 class I histocompatibility antigen, AA alpha chain-like |
| 39 | XM_015991682.1 | H-2 class I histocompatibility antigen, D-37 alpha chain-like |
| n/a | XM_006997716.2 | Class I histocompatibility antigen, Gogo-A*0201 alpha chain-like |
| n/a | XM_006996074.1 | HLA class I histocompatibility antigen, A-36 alpha chain-like |
| n/a | XM_006999020.1 | Class I histocompatibility antigen, Gogo-A*0401 alpha chain-like |
| n/a | XM_006993083.2 | Regulatory factor X1 (Rfx1), transcript variant X1, mRNA |
| n/a | XM_006996080.2 | HLA class I histocompatibility antigen, A-29 alpha chain-like |
| n/a | XM_006997675.1 | H-2 class I histocompatibility antigen, Q10 alpha chain-like |
| n/a | XM_015990691.1 | HLA class I histocompatibility antigen, A-32 alpha chain-like |

| **Table S3.** Enrichment of gene ontology (GO) categories of genes differentially expressed between the two polygynandrous species. Data are from 6,435 genes that were differentially expressed between the two polygynandrous study species. GO categories examined were cellular component, biological process, and molecular function. In all cases, enrichment was significant at p < 0.05 after correction with the Benjamini-Hochberg ‘BH’ procedure. Shaded entries denoted GO categories containing genes that were enriched in both species | | | |
| --- | --- | --- | --- |
| **Up-regulated in *P. boylii*** | | **Up-regulated in *P. maniculatus*** | |
| **Term** | | **Term** | |
| **GO ID** | **Cellular Component** | **GO ID** | **Cellular Component** |
| GO:0005615 | extracellular space | GO:0005615 | extracellular space |
| GO:0009897 | external side of plasma membrane | GO:0009897 | external side of plasma membrane |
| GO:0016021 | integral component of membrane | GO:0016021 | integral component of membrane |
| GO:0005887 | integral component of plasma membrane | GO:0005576 | extracellular region |
|  |  | GO:0031090 | cell surface |
|  |  | GO:0031090 | organelle membrane |
| **Biological Process** | | **Molecular Function** | |
| GO:0045087 | innate immune response | GO:0005506 | iron ion binding |
| GO:0051607 | defense response to virus | GO:0005509 | calcium ion binding |
|  |  |  |  |


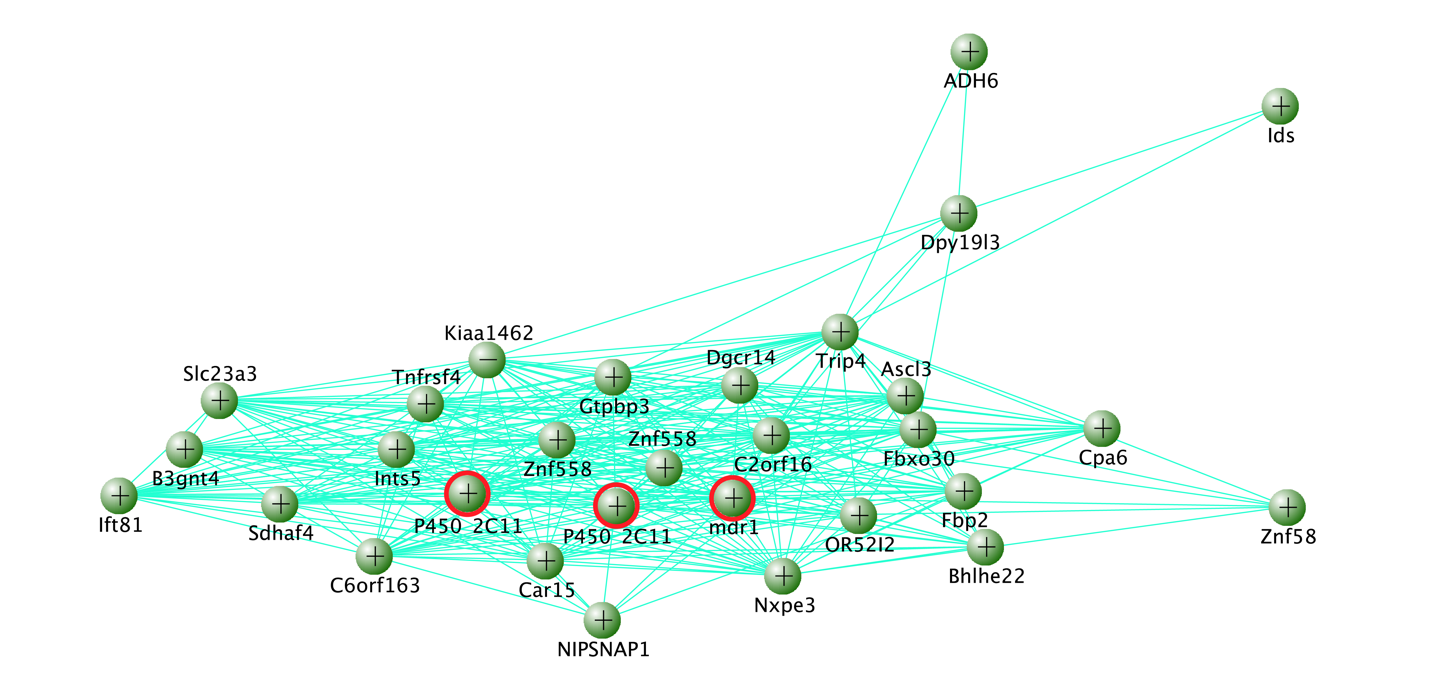


**Figure S1.** Gene network for the top most connected genes (N = 30) in the brown module. Only connections between genes with a topological overlap of greater than 0.12 are shown. Genes with known important immune system functions are highlighted with a red circle.

**Figure S2.** Functional classifications of genes assigned to the brown expression module. Function was determined for 523 genes based on gene ontology (GO) category assignments; the percentage of genes assigned to each GO category is shown. In (A), data for genes corresponding to molecular function GO terms are shown; in (B), data for genes corresponding to biological process GO terms are shown.
